# Psychometric properties and measurement invariance of the Beck hopelessness scale (BHS): results from a German representative population sample

**DOI:** 10.1186/s12888-018-1646-6

**Published:** 2018-04-25

**Authors:** Sören Kliem, Anna Lohmann, Thomas Mößle, Elmar Brähler

**Affiliations:** 1 Criminological Research Institute of Lower Saxony, Lützerodestraße 9, 30161 Hannover, Germany; 2 State Police College of Baden-Wuerttemberg, Sturmbühlstraße 250, 78054 Villingen-Schwenningen, Germany; 3 Department of Psychosomatic Medicine and Psychotherapy, University of Mainz, |Untere Zahlbacher Str. 8, 55131 Mainz, Germany; 4 Department of Medical Psychology and Medical Sociology, University of Leipzig, Philipp-Rosenthal-Str. 55, 04103 Leipzig, Germany

**Keywords:** Beck hopelessness scale (BHS), Hopelessness, Population sample, Measurement invariance, Psychometric analysis, Confirmatory factor analysis (CFA), Bi-factor model, Factor structure

## Abstract

**Background:**

The Beck Hopelessness Scale (BHS) has been the most frequently used instrument for the measurement of hopelessness in the past 40 years. Only recently has it officially been translated into German. The psychometric properties and factor structure of the BHS have been cause for intensive debate in the past.

**Methods:**

Based on a representative sample of the German population (*N* = 2450) item analysis including item sensitivity, item-total correlation and item difficulty was performed. Confirmatory factor analyses (CFA) for several factor solutions from the literature were performed. Multiple group factor analysis was performed to assess measurement invariance. Construct validity was assessed via the replication of well-established correlations with concurrently assessed measures.

**Results:**

Most items exhibited adequate properties. Items #4, #8 and #13 exhibited poor item characteristics– each of these items had previously received negative evaluations in international studies. A one-dimensional factor solution, favorable for the calculation and interpretation of a sum score, was regarded as adequate.

A bi-factor model with one content factor and two method factors (defined by positive/negative item coding) resulted in an excellent model fit. Cronbach’s alpha in the current sample was .87. Hopelessness, as measured by the BHS, significantly correlated in the expected direction with suicidal ideation (*r* = *.*36), depression (*r* = *.*53) and life satisfaction (*r* = −*.*53). Strict measurement invariance could be established regarding gender and depression status. Due to limited research regarding the interpretation of fit indices with dichotomous data, interpretation of CFA results needs to remain tentative.

**Conclusion:**

The BHS is a valid measure of hopelessness in various subgroups of the general population. Future research could aim at replicating these findings using item response theory and cross-cultural samples. A one-dimensional bi-factor model seems appropriate even in a non-clinical population.

**Electronic supplementary material:**

The online version of this article (10.1186/s12888-018-1646-6) contains supplementary material, which is available to authorized users.

## Background

Hopelessness as a psychological construct is of relevance with regard to various psychological disorders and related symptoms, e.g. depression, suicide, schizophrenia, alcoholism and sociopathy. Due to its role in the etiology of depression, hopelessness became a focus of the work group around Aaron T. Beck. In his cognitive theory, Beck conceptualizes hopelessness as a system of cognitive schemes. These schemes can be characterized by a generalized negative future expectation. An individual characterized as hopeless overestimates the likelihood of unfortunate events while underestimating the occurrence of fortunate and positive events. Positive outcomes are regarded as being very unlikely if not impossible [1]. These dysfunctional beliefs regarding the future are a cornerstone of Becks cognitive triad of depression [2]. The other two elements of this triad are a negative view of the self and a negative view of the world. Although hopelessness and depression correlate very highly, and hopelessness is often observed in depressed individuals, it is not a necessary component of depression [3]. This is taken into account in Abramson’s theory of *hopelessness depression*, a special subtype of depression. Hopelessness is a proximal cause of the symptoms of hopelessness depression and suicidality is a symptom of this special form of depression, however not of other forms of the clinical picture of depression [3].

In over 30 years of research, Beck and his colleagues established that hopelessness is more strongly related to suicidality than to depression [4]. In their cognitive model of suicidal behavior Wenzel and Beck [5] acknowledge the crucial role of hopelessness in the context of suicide. They place cognitive schemes at the center of suicidal behavior, one of these being state hopelessness. Furthermore, the World Health Organization [6] recognizes hopelessness as an important suicide risk factor and recommends its assessment in the context of suicidal behavior. Besides its crucial role in suicidality, hopelessness is an important construct in the context of life satisfaction, compliance and recovery in medical care, as well as in the field of forensic psychology [7, 8].

Since its development in 1974, the Beck Hopelessness Scale (BHS) has become the most popular measurement of the hopelessness construct in international studies. It has been translated into dozens of languages and used in hundreds of studies worldwide. The BHS is a 20 item self-assessment questionnaire. All items are scored on a true-false rating scale. After recoding negatively worded items, the number of endorsed items is combined to a sum-score. Based on their own research the authors of the English original suggest the following classification of the BHS-sum score: 0–3 minimal, 4–8 mild, 9–14 moderate, 15–20 severe. Based on investigations on the predictive power of the BHS, most researchers investigating the predictive power of the BHS suggest a cut-off of 9 to be indicative of suicide intentions [4, 9–11]. However, final interpretations should be left to trained clinicians [12].

Although the BHS is well established it had not officially been translated into German. Furthermore, despite this widespread use in various different samples, measurement invariance and hence the appropriateness of intergroup comparison in different populations, has never been tested. Hence, the aim of the present study was to assess the validity, reliability, factor structure, construct validity and factorial invariance of the German version of the BHS. Data stems from a sample representative for the population of the Federal Republic of Germany. To the knowledge of the authors only four other studies report the use of the BHS in a population sample [13–16]. Only Tanaka and colleagues report psychometric characteristics of the scale for a Japanese sample of *N* = 154 community residents. Psychometric properties of the scale have so far not been reported for a large western representative community sample. (Iliceto and Fino [16] have used the scale in a large Italian population, however the sample was not representative. Furthermore, the researchers used the BHS with a 5-point Likert format.)

Szabó and colleagues [17] recently investigated a bi-factor model with one content related and two method-factors in a clinical population from Hungary. Boduszek and Dhigra [18] have recently investigated the factor structure in a large student sample building on the work of Szabó and colleagues [17] suggesting a three dimensional multi trait multi method model (MTMM). In addition to replicating the results from other large European studies and validating the German version of the BHS, this study is furthermore the first to combine the recently favored methodological approach (i.e. using method factors accounting for item wording using a bi-factor or MTMM framework) with the analysis of measurement invariance in a large representative population sample.

## Method

### Sample and sampling procedure

From February to June 2014 the University of Leipzig conducted a survey in a population sample representative of the Federal Republic of Germany. A total of *N* = 2527 individuals were interviewed by 206 interviewers. This number corresponds to response rate of 54.8% of the initially contacted households (*N* = 4607). The interviews were conducted by professionals from an independent institute for opinion and social research (USUMA Berlin). The sampling was carried out using a threefold random selection procedure drawing from in the entire inhabited territory of the Federal Republic of Germany. In a first step, 258 non-overlapping regional areas in Germany were defined by use of Cox-allocations. After this, target households were randomly selected within these areas through random route procedures. Finally, the interviewers identified the target person within the household with the help of a Kish selection grid [19]. Each target person was individually interviewed at home by a trained interviewer and was asked to complete several self-report questionnaires. The proper conduct of the interviews was controlled. For this purpose, postcards with pre-payed postage were sent to 38.7% of the participants. Approximately 53% of these postcards were returned, all of them confirmed that the interviewers had worked as expected. As indicated in the original BHS manual, the BHS was only presented to participants who were at least 18 years old. Hence, a subsample of 2450 individuals is the basis of the present study. The participant’s mean age was *M* = 50.51 years (*SD* = 17.0) with a range of 18–95 years; 88 (3.6%) had nationalities other than German; 54% were female. All participants had adequate knowledge of the written German language and completed the survey entirely in German. Further sample details can be obtained from Table 1. Written informed consent was obtained from each participant. The survey was approved by the ethics committee of the medical faculty of Leipzig University (AZ: 063–14-10,032,014).

**Table 1:** Demographic characteristics of the study sample

Sample characteristics	Men	Women	Total sample
	(*N* = 1130)	(*N* = 1320)	(*N* = 2450)
Age group, *N* (%)			
18–24	88 (7.8%)	93 (7.0%)	181 (7.4%)
25–34	150 (13.3%)	188 (14.2%)	338 (13.8%)
35–44	183 (16.2%)	218 (16.5%)	401 (16.4%)
45–54	221 (19.6%)	264 (20.0%)	485 (19.8%)
55–64	225 (19.9%)	251 (19.0%)	476 (19.4%)
65–74	177 (15.7%)	183 (13.9%)	360 (14.7%)
> 74	86 (7.6%)	123 (9.3%)	209 (8.5%)
Living with a partner, *N* (%)	702 (63.1%)	740 (57.2%)	1442 (59.9%)
Having at least 1 child, *N* (%)	208 (18.4%)	341 (25.8%)	549 (22.4%)
Member of a church, *N* (%)	770 (68.5%)	984 (74.8%)	1754 (71.9%)
Level of education attained, *N* (%)			
Completed Year 9	429 (38.0%)	454 (34.4%)	883 (36.0%)
Completed Year 10	412 (36.5%)	573 (43.5%)	985 (40.2%)
Completed Year 12	118 (10.4%)	120 (9.1%)	238 (9.7%)
University Degree	133 (11.8%)	122 (9.2%)	255 (10.4%)
Other	38 (3.4%)	51 (3.9%)	89 (3.5%)
Employment status, *N* (%)			
In Training	48 (4.3%)	47 (3.5%)	95 (3.8%)
Working (> 35 h)	605 (53.8%)	388 (29.5%)	993 (40.7%)
Working (< 35 h)	51 (4.5%)	316 (24.0%)	367 (15.0%)
Unemployed	74 (6.6%)	77 (5.9%)	151 (6.2%)
Homemaker	10 (0.9%)	93 (7.1%)	103 (4.2%)
Retired	334 (29.7%)	377 (28.6%)	711 (29.1%)
Other	2 (0.2%)	18 (1.4%)	20 (0.8%)
Missing	6 (0.5%)	4 (0.3%)	10 (0.4%)
Monthly household income in €, *N* (%)			
< 1250	168 (14.9%)	291 (22.0%)	459 (18.7%)
1250–2000	299 (26.5%)	365 (27.7%)	664 (27.1%)
> 2000	633 (56.0%)	630 (47.7%)	1263 (51.6%)
Missing	30 (2.7%)	34 (2.6%)	64 (2.6%)

### Questionnaires

#### The Beck hopelessness scale (BHS)

The BHS [20] is a 20 item self-assessment instrument for the measurement of hopelessness. Abbreviated items are presented in Table 2. The respondent is asked to evaluate each of the 20 statements and decide whether the statement describes his or her attitude in the previous week (including the day of assessment). Nine items are inversely scored to prevent acquiescence. After inversion of the positively worded items, a sum-score is calculated. The total score can range from 0 to 20, indicating the number of items endorsed in the hopelessness direction. Translation of the scale into German was carried out by Pearson Assessment on the basis of WHO guidelines for questionnaire translation (including forward and back-translations). Results from the same survey using the German BHS and BSS have also been published by Gunzelmann and colleagues [21] in German .

**Table 2:** Item characteristics

Item	Item content	Endorsement rate	SE	*P*_*i*_	*r*_*it*_
1^*^	hope and enthusiasm	0.19	0.008	19	.56
2	might as well give up	0.13	0.007	13	.49
3*	bad things won’t stay forever	0.08	0.005	8	.33
4	can’t imagine live in 10 years	0.51	0.010	51	.25
5*	enough time for accomplishments	0.21	0.008	21	.48
6*	expect to succeed	0.13	0.007	13	.57
7	dark future	0.14	0.007	14	.64
8*	particularly lucky	0.55	0.010	55	.20
9	can’t get the breaks	0.25	0.009	25	.46
10*	well prepared for the future	0.10	0.006	10	.45
11	unpleasantness ahead	0.19	0.008	19	.64
12	don’t expect to get what I really want	0.35	0.010	35	.50
13*	future will be happier	0.52	0.010	52	.03
14	things just don’t work out	0.31	0.009	31	.57
15*	faith in the future	0.21	0.008	21	.57
16	never get what I want	0.13	0.007	13	.53
17	real satisfaction unlikely	0.18	0.008	18	.63
18	future vague and uncertain	0.30	0.009	30	.59
19*	more good times	0,19	0.008	19	.53
20	no use in trying	0.21	0.008	21	.56
		M	SD	*P*_*i*_	
BHS-sum score		4.87	4.33	24	–

*Note.* Items marked by an asterisk indicate inversely scored items. All correlations were significant on the .001 level; *SE* = Standard Error of the endorsement rates; *P*_i_ = item difficulty; *r*_it_ = item-rest correlation

#### The Beck scale for suicide ideation (BSS)

The BSS [22] contains 21 statement groups each consisting of three sentences that differ in the intensity of suicidal ideation. Scores between 0 and 2 are designated to each statement. Participants chose one statement of each group, which describes them best. The total BSS score can range from 0 to 38, with higher values indicating an increasing risk of suicide. The first five items of the BSS serve as a screening tool for suicidal ideation during the previous week and can be summed up to form the BSS-Screen score. Subsequent items are only presented if either item # 4 or item #5 has been endorsed. These 14 items allow for an assessment of the severity of the existing suicidal ideation. The last two statement groups address frequency and intensity of former suicide attempts and are to be answered by all participants independent of their endorsement of the filter questions. They are however not included in the total BSS score. Due to the low number of individuals that have completed the whole questionnaire, only the screening part of the BSS will be used for the establishment of construct validity. Details on the psychometric properties of the German version of the BSS can be obtained from Kliem and colleagues [23].

#### The patient health questionnaire 2 (PHQ-2)

The PHQ-2 is a brief instrument for the assessment of depressive symptoms. It consists of only two items, namely the depression scale of the PHQ-9 [24]. It assesses the frequency of depressive symptoms over the course of the previous 2 weeks. The response options for each item are 0 = not at all, 1 = several days, 2 = more than half the days and 3 = nearly every day. Thus, PHQ-2 scores can range from 0 to 6. A total score of 3 proved to be most suitable regarding sensitivity and specificity for the tentative diagnosis of major depressive disorder (sensitivity: 87%, specificity: 78%) as well as other depressive disorders (sensitivity: 79%, specificity: 86%). Cronbach’s alpha was found to be α = .83 [25].

#### Life satisfaction questionnaire (FLZ)

The FLZ-8, a shortened version of the Fragebogen zur Lebenszufriedenheit (FLZ) [Life Satisfaction Questionnaire] by Brähler, Fahrenberg, Myrtek, and Schumacher [26], was used for the assessment of global life satisfaction. It assesses individual satisfaction in eight areas of life (friends/acquaintances, leisure time/hobbies, health, income/financial security, job/work, living situation, family life/children, relationship/sexuality). Participants have to rate their satisfaction in each area on a 5-point rating scale ranging from 1 = dissatisfied to 5 = very satisfied. Individual items can be aggregated to form a global score. The sum score can range from 7 to 35 with higher values indicating higher life satisfaction. Cronbach’s alpha for the original sub-scales ranged from α = .82 to α = .95 [26].

### Imputation of missing data

A percentage of 0.0–1.4% of the data were missing on item level regarding the questionnaires examined in the context of this paper. Missing data were imputed using nonparametric recursive partitioning. The R package missForest [27] was used for this purpose. Differing from other established imputation methods (e.g., multiple imputation or full information maximum likelihood) the missForest imputation algorithm is not based on any assumptions regarding the distribution underlying the estimated variables, which makes it particularly suitable for mixed-type data containing variables differing in level of measurement. Non-linear relationships as well as higher order interactions are more adequately modeled this way. The random forest algorithm constructs a multitude of decision trees based on the observed values. After being trained on the data this way, in a next step the missing values are predicted for each variable on the basis of the other variables of the data set. This process is undergone for each variable and then repeated iteratively until a stopping criterion is met. The random forest was trained based on the variables age, gender, BHS items and BSS-Screen items. The FLZ-8 and the PHQ-2 were not imputed on the item level as the original survey data available to the authors only included the sum score, hence only the sum score could be imputed if missing.

### Psychometric analysis

The BHS is dichotomously-scored and hence can be categorized as an ordered-categorical measure whose values are both discrete and ordinal in scale. Ordered-categorical measures require some special considerations. For example, a dichotomous variable can by definition never be normally distributed. Hence, every method based on this prerequisite is not applicable. Whenever properties at the item level are assessed, special attention has to be given to methodology.

#### Internal consistency reliability

Reliability for dichotomous measures can be computed using the Kuder-Richardson Formula 20 (KR-20). The KR-20 is analogous to Cronbach’s alpha which is a generalization of the same for non-dichotomous measures.

#### Item analysis

In dichotomous items, item difficulty is calculated as the percentage of individuals who endorsed the item, after recoding all variables such that endorsement indicates higher levels of hopelessness. Item-total correlations refer to the correlation of a single item with the rest of the scale.

#### Factorial validity

CFA was performed using the lavaan package [28] for R statistics. As suggested for the use with ordered categorical measures [29], weighted least square means and variance adjusted estimation (WLSMV) was used. Model-fit was assessed using the following fit measures: Comparative Fit Index (CFI), Tucker Lewis Index (TLI), Root Mean Square Error of Approximation (RMSEA). It is common practice to use the same criteria for evaluating goodness of fit for ordered categorical measures as for continuous ones, with the canonical reference being Hu and Bentler [30]. While some authors suggest this procedure for lack of alternative (e.g. [31]) adequacy of this procedure has not yet been established. As χ^2^ has been found to be overestimated even with robust WLS estimation interpretation of model fit will not rely too heavily on χ^2^ estimation. In this paper, for the lack of alternatives, we will therefore rely on the Hu and Bentler [30] criteria of a CFI and TLI > .95 and a RMSEA < .08 indicating good model fit. Robust test statistics were used. However, due to the lack of validation of this interpretation in the context of WLSMV estimation of ordered categorical variables, especially dichotomous ones, all interpretation of model fit has to remain tentative.

First we analyzed, the original model by Beck and colleagues [20] [a three-factor model: Feelings, about the future (1,5,6,13,15,19), Loss of motivation (2,3,9, 11,12,16,17,20), Future Expectations (4,7,8,10,14,18)] and the one-dimensional model (all items loading on one overall hopelessness factor) as these are the models most discussed in the BHS literature. Recently the influence of method factors (optimism and pessimism, due to item wording) has been introduced in the discussion regarding the BHS factor structure. Therefore, we also tested a one-factorial, bi-factor model as suggested by Szabó and colleagues [17] [one content factor (all items), two method factors: Negatively worded items (2,4,7,9,11,12, 16, 17, 18, 20) and Positively worded items (1,3,5,6,8,10,13,15,19)] as well as a multitrait-multimethod model suggested by Boduszek & Dhingra [18] [three correlated trait factors (as suggested by Beck and colleagues), and two correlated method factors] .

Based on the fact, that the factor structure of the BHS has been fiercely debated and various factor solutions were suggested by different research groups, we additionally tested several other models suggested in the BHS literature. Details regarding those models can be found in the Additional file 1 . Especially noteworthy are the following three models, which will thoroughly be reported in the results section and critically evaluated in the discussion section.

However, the BHS sum-score is the major statistic that is interpreted in every day practice. Uni-dimensionality can hence be regarded as a mayor objective. To justify the endorsement of a three-factorial solution instead of a more parsimonious one-factorial model, the sub-factors have to add explanatory value (for example differential predictive validity; see [18]). To examine differential relationships exhibited between the three conceivable hopeless factors (“feelings about the future”, “loss of motivation”, and “future expectations”) correlations with the following constructs were computed: life satisfaction (FLZ), suicidal ideation (BSS-Screen), depression (PHQ-2), as well as two single items indicating death wish and suicide attempt.

To further test the reliability of the bifactor model (one general factor) as well as the MTMM model (three-factors). Mc Donald’s coefficient omega (ω) was computed for each of the subscales as well as the overall sum score.

#### Measurement invariance

Measurement invariance can be assumed if a construct’s factorial structure does not differ between groups (e.g. gender), i.e., the factor structure is invariant across these groups. There are different levels of measurement invariance, which determine the comparability of the analyzed groups. Configural invariance, the weakest form of measurement invariance, refers to the equivalence of the factorial structure. It is assumed when the latent constructs (i.e., the factors) show the same dimensionality and in addition, the indicators (i.e., the items) can be identically assigned to the latent constructs in both groups. This type of measurement invariance is necessary but not sufficient for allowing an unbiased comparison of measurement between groups. If this prerequisite is not empirically supported, stricter tests of invariance and comparisons between groups are not appropriate, as this indicates that the analyzed items measure different constructs in the examined groups.

More restrictive forms of measurement invariance refer to a) the factor loadings (i.e., metric or weak invariance) and b) the intercepts of the indicators (i.e., scalar or strong invariance). If weak invariance is empirically supported, structural relationships among latent constructs (e.g., correlation coefficients) can be compared between the groups. If, additionally, strong invariance is also confirmed, between-group differences in the constructs’ means can be assessed. Ultimately, error variance invariance (or strict invariance) can be tested examining whether the indicators’ residual variances are equal across groups. Different residual variances in groups can result in different error rates (e.g., sensitivity, specificity) and thereby affect screening decisions or the calculation of critical differences (e.g., [32]) Measurement invariance was assessed based on the following groups: gender (as indicated by participants) and depression (PHQ-2 score below 3, PHQ-2 score of 3 or higher). Categorization of depression status reflects the suggested cut-off for a tentative diagnosis of depressive illness [25]. Measurement invariance was tested using multiple group factor analysis, which was again performed using the lavaan package [28] for R statistics and WLSMV estimation. Following the procedure suggested by Millsap and Yun-Tein [33] for ordered categorical variables, the following models were subsequently tested: configural invariance (no constraints apart from those necessary for model identification), weak invariance (constraining all loadings to be equal), strong invariance (constraint of threshold which was already necessary for model identification and is hence identical to weak invariance), strict invariance (constraining unique variances to 1). Chen [34] suggest the following cut-off criteria: a change of ≥ − .01 in CFI in addition to a change of ≥.015 in RMSEA indicates non-invariance. They furthermore point out that among those indices CFI is the most reliable and that RMSEA tends to be more affected by sample size and model complexity.

#### Construct validity

Construct validity was established by assessing the correlations of the BHS sum score and the Fragebogen zur Lebenszufriedenheit (FLZ-8) [Life satisfaction questionnaire] and a brief assessment of depressive symptoms: Patient Health Questionnaire (PHQ-2) as well as the Beck Scale for Suicide Ideation (BSS).

## Results

Sample characteristics including descriptive statistics of demographic variables can be obtained from Table 1 in the methods section.

### Item characteristics

Table 2 presents the BHS item characteristics. BHS mean in the whole sample was *M* = 4.87 (*SD* = 4.33). Item difficulty *P*_*i*_ ranged from 8 (# 3 “bad things won’t stay forever”) to 55 (#8 “particularly lucky”). Item-total correlations ranged from *r*_*it*_ = .03 (#13 “future will be happier”) to *r*_*it*_ = .64 (#7 “dark future”). Item means were virtually identical between men and women with effect sizes between *d* = .00 and *d* = .09.

### Factor structure

Confirmatory factor analysis of the one-dimensional model exhibited an acceptable model fit χ^2^ = 2205.127***, df = 170, CFI = 0.926, TLI = 0.917, RMSEA = 0.070, 95% CI [0.067, 0.073]. For a bi-factor model accounting for the optimism and pessimism method factors recently suggested by Szabó and colleagues [17] excellent model fit could be attested (χ^2^ = 562.577***, df = 150, CFI = 0.985, TLI = 0.981, RMSEA = 0.034, 95% CI [0.031, 0.036]). Composite reliability in the form of McDonald’s coefficient omega was ω = 0.90 (for the general factor). Standardized factor loadings for this model can be obtained from Table 3.

**Table 3:** Standardized factor loadings from CFA of orthogonal bi-factor model specifying one content-related factor and two method factors (depending on item-coding) as well as a general one-factor model

		Bi-factor model			Unidimensional model
Item	Item content	Factor 1 (content)	Factor 2(method pos.)	Factor 2(method neg.)	General factor
1*	hope and enthusiasm	0.686	0.515	–	0.769
2	might as well give up	0.674	–	0.403	0.751
3*	bad things won’t stay forever	0.563	0.217	–	0.572
4	can’t imagine live in 10 years	0.358	–	0.167	0.377
5*	enough time for accomplishments	0.583	0.504	–	0.671
6*	expect to succeed	0.733	0.519	–	0.815
7	dark future	0.910	–	0.116	0.878
8*	particularly lucky	0.195	0.516	–	0.292
9	can’t get the breaks	0.588	–	0.438	0.678
10*	well prepared for the future	0.652	0.404	–	0.706
11	unpleasantness ahead	0.850	–	0.263	0.870
12	don’t expect to get what I really want	0.633	–	0.390	0.705
13*	future will be happier	−0.081	0.488	–	0.034
14	things just don’t work out	0.770	–	0.262	0.793
15*	faith in the future	0.697	0.476	–	0.765
16	never get what I want	0.668	–	0.593	0.825
17	real satisfaction unlikely	0.797	–	0.409	0.871
18	future vague and uncertain	0.861	–	0.020	0.800
19*	more good times	0.681	0.385	–	0.725
20	no use in trying	0.646	–	0.636	0.812

*Note:* Items marked by an asterisk indicate inversely scored items

For several multi-factorial models (e.g. the model suggested by Beck and colleagues [20]: Feelings, about the future (1,5,6,13,15,19), Loss of motivation (2,3,9, 11,12,16,17,20); Future Expectations (4,7,8,10,14,18), χ^2^ = 1499.105 ***; df = 167; CFI = 0.951; TLI = 0.945, RMSEA = 0.057, 95% CI [0.054, 0.060]) acceptable to good model fit could be confirmed.^1^ Excellent model fit was obtained for the MMTM model recently suggested by Boduszek and Dhingra [18] with three correlated traits and two correlated method factors (χ^2^ = 474.106***, df = 146, CFI =0.988, TLI =0.984, RMSEA = 0.030, 95% CI [0.027, 0.033]). Composite reliability in the form of McDonald’s coefficient omega was ω = 0.96 (for total score), ω = 0.88 (factor 1 - Feelings about the future), ω = 0.94 (factor 2 - Loss of motivation) and ω = 0.90 (factor 3 - Future expectations).

To evaluate rationale regarding the different models we investigated whether the original Beck factors (as also included in the MTMM model) provide additional explanatory value regarding construct validity. Table 4 shows the correlations of the three BHS factors as originally suggested by Beck and colleagues [20]. As the correlations only marginally differ per factor we continued further analyses using one-dimensional approach.

**Table 4:** Correlations of the BHS-Score and BHS factors with theoretically relevant constructs

Variable	*M*	*SD*	1	2	3	4	5	6	7	8
1. BHS	4.87	4.33	–	–	–	–	–	–	–	–
2. Feelings about the future	1.44	1.57	.78	–	–	–	–	–	–	–
3. Loss of motivation	1.52	2.05	.87	.46	–	–	–	–	–	–
4. Future expectations	1.91	1.54	.86	.56	.63	–	–	–	–	–
5. PHQ-2	0.94	1.18	.53	.37	.48	.47	–	–	–	–
6. FLZ-8	29.25	6.09	−.53	−.40	−.43	−.52	−.51	–	–	–
7. BSS-Screen	0.22	0.98	.36	.27	.33	.29	.33	−.27	–	–
8. Suicide attempt	0.05	0.21	.35	.27	.32	.29	.30	−.25	.76	–
9. Death wish	0.05	0.25	.30	.24	.27	.25	.31	−.25	.87	.56

*Note.* All correlations are Pearson coefficients. PHQ-2 = Patient Health Questionnaire 2-item short form; FLZ = life satisfaction questionnaire 8-item short form; BSS-Screen = 5 screening items of the Beck Scale for Suicide ideation. All *p* < .001. (two-tailed)

### Measurement invariance

Table 5 shows the fit measures obtained in the measurement invariance analysis for the one-factorial as well as for the bi-factor model. The cut-off criteria by Chen [34] are exceeded only in the strict bi-factor model regarding depression status. Robust fit statistics are reported. The groups were of the following sizes. Gender: female *n* = 1320, male *n* = 1130; depression status: non-depressed *n* = 2227, possibly depressed *n* = 223.

**Table 5:** Results of the Multi Group Confirmatory Factor Analysis

	Model	*X* ^*2*^	*df*	*CFI*	*RMSEA*	95% CI	Δ*CFI*	Δ*RMSEA*
	Group = Gender							
One factor	Configural	1.635.501	238	0.945	0.063	[0.060,0.066]	–	–
	Weak / strong	1.625.190	254	0.948	0.066	[0.063,0.070]	0.003	0.003
	Strict	1.480.973	270	0.954	0.061	[0.058,0.064]	0.006	−0.005
bi-factor	Configural	682.7209	299	0.986	0.032	[0.029,0.036]	–	–
	Weak / strong	711.6125	336	0.986	0.030	[0.0278,0.033]	< 0.001	−0.002
	Strict	674.5467	354	0.989	0.027	[0.024,0.030]	0.003	−0.003
	Goup = Depression (PHQ-2 score < 3, PHQ-2 score ≥ 3)							
One factor	Configural	1.613.255	238	0.927	0.069	[0.066,0.072]	–	–
	Weak / strong	1.618.950	254	0.927	0.066	[0.063,0.069]	< 0.001	−0.003
	Strict	1.520.225	270	0.933	0.062	[0.059, 0.065]	0.006	−0.004
bi-factor	Configural	673.1354	299	0.981	0.032	[0.029, 0.035]	–	–
	Weak / strong	1023.4319	336	0.965	0.041	[0.038, 0.044]	−0.016	0.009
	Strict	961.7185	354	0.969	0.037	[0.035, 0.040]	0.004	−0.004

*Note.* All fit statistics are robust.; configural = (for identification purposes) one marker variable per factor fixed to 1, unique variances of marker variables fixed as 1; all thresholds equally constrained across groups, unique variance of first group fixed as 1, factor means of first group fixed as 0; weak/strong = additionally all free loadings constrained to be equal across group; strict = additionally all unique variances of all groups fixed to 1; *CFI* = Comparative Fit Index, *RMSEA* = Root Means Square Error of Approximation, *** = *p* < .001

### Construct validity

Table 4 contains the Pearson correlations of the BHS sum scores and measures of depression (PHQ-2) suicidal ideation (BSS-Screen) and life satisfaction (FLZ-8). The directions of the correlations were in accordance with theoretical expectations.

## Discussion

This study examined the psychometric properties of the German version of the Beck Hopelessness Scale in a large sample representative for the Federal Republic of Germany. It has been the first study to report psychometric properties of the BHS in a large representative western community sample and the first investigations of the scales measurement invariance. Although several authors attest limited suitability of the BHS for general population samples [7, 35] the German version of the Beck Hopelessness Scale demonstrated mostly sound psychometric properties. The item characteristics can be evaluated as satisfactory with a few noteworthy exceptions: The items #4, #8 and #13 cannot be interpreted as adequately capturing the construct. In previous psychometric evaluations these specific items have also been found to be the cause for concern. Niméus et al. [11] performed a principal component analysis and found item #4 to load on a single factor all on its own. Kao, Liu, and Lu [36] found that item #4 was endorsed 1.5 times more frequently in non-suicidal patients than in suicidal patients. No other item showed such inverse behavior, at least not to that extent. A similar item behavior was observed by Durham [7] who found that item #13 had the same item difficulty in a student sample as in psychiatric samples. In the following studies, one or more of the problematic items exhibited low item-total correlations or very low factor loadings: Aloba, Ajao, Alimi and Esan [37], Fisher and Overholser [38], Perczel Forintos, Sallai and Rózsa [39], Pompili, Tatarelli, Rogers and Lester [40], Steed [41], Szabó et al. [17], Tanaka, Sakamoto, Ono, Fujihara and Kitamura [15], Young, Halper, Clark, and Scheftner [35]. However, the undesired properties of these items are not universal. In fact, in some studies they ranged among the best. In the Yoruba version of the BHS, Aloba et al. [42] even suggested two of these items (namely #8 and #13) for inclusion in a 4-item short form. Tanaka et al. [15] reports negative item-total correlation of item #17 in a Japanese population sample, this item ranged among the best in the German sample, suggesting potential cultural differences. The zeitgeist however should also be considered. The BHS has been developed in the 1970s. Especially the items that turned out to be problematic in this analysis, could be interpreted as historically sensitive. Being able to imagine one’s life in 10 years (item #4) is probably harder in 2018 than it was in 1974 (at least in Germany). Individuals from first world countries today more frequently change careers, remarry or move houses; which does not necessarily render them more hopeless. Viewing oneself as particularly lucky (item #8) might be rooted in the notion of fate, a concept that was arguably more pronounced in the 1970s. Expecting to be happier in the future (item #13) possibly highly depends on the current situation. Anyone being currently very happy might also have a hard time imagining an even more positive future. This notion is supported by a recent study by Szabó and colleagues [17] who report, that item #13 tapped into both optimism as well as pessimism. The current state of happiness might also fluctuate with recent historic events.

### Factorial validity

Confirmatory factor analysis of the one-factor model provided acceptable model fit. This allows the calculation (and meaningful interpretation) of a BHS sum score. When including two method factors (one for positively worded items and one for negatively worded items) model fit of the one-dimensional bi-factor model as suggested by Szabó et al. [17] results in excellent model fit for the one-factor solution.

Regarding multi-factorial models, most of the three factor solutions (e.g. Beck [20]) fit the data well. When taking into account the partially inverted item wording, the fit of the three factor MTMM model suggested by Boduszek and Dhingra [18] was almost identical excellent as the bifactor model. However, given that we could not reproduce differential relationships exhibited between the three hopelessness factors, we would opt for the more parsimonious one-dimensional bi-factor model. Furthermore, some methodological arguments support the reasoning that the good fit of several three factorial models might be due to method effects. For example, Woods [43] pointed out that dichotomous items tend to produce spurious factors, as items with a similar threshold tend to group together in factors. Thus, the good fitting three factor solutions could in fact be an artifact. The notion of Flora and Curran [29], that positive bias of several fit measures increase with model size using WLSMV estimation, is another explanation for the good fit of several models. A final recommendation however cannot be provided. Further studies depending on large samples seem necessary to reach a final conclusion. It seems furthermore possible that different factorial solutions are to be favored in different sample compositions. Several BHS publications have discussed the notion that in non-clinical samples hopeless is best interpreted as a unidimensional construct, whereas in clinical samples a multi-factorial approach is more appropriate (since a certain degree of hopelessness is necessary to bring out the differences). This could also be an explanation regarding the different explanatory value of the subfactors in the present study compared to the recent study by Boduszek and Dhingra [20] as the BHS mean in their student sample is considerably higher than in the present sample.

### Measurement invariance

Measurement invariance analysis using multiple-group CFA supported invariance across gender and depression status. Cultural explanations however could not be tested and should be taken into account in future research. Cross cultural assessment using the BHS is scarce (the authors know of only one study including American and Turkish university students, [44] and therefore the establishment of measurement invariance in cross cultural samples might be an adequate next step.

### Construct validity

Construct validity could be established by replicating correlations from the existing body of research. Correlations of hopelessness and depression (measured with the PHQ-2) are comparable in magnitude to those found in previous research with various measures of depressive symptoms. However, it should be noted that the second of the two PHQ-2 items explicitly assesses feelings of hopelessness, which could possibly inflate the correlation. The correlation of the BHS with the measure of life satisfaction (FLZ) are in the expected direction and exceed previously found correlations in magnitude. Correlations with suicide related measures (BSS-screen, suicide attempt and death wish) are highly significant but lower than expected with regard to previous findings. This might be due to the unusually small percentage of suicidal individuals given the otherwise large sample size. One of the most relevant characteristic of the hopelessness construct, namely the fact that hopelessness stronger correlates with suicidality than depression correlates with the same, is not so pronounced in this sample. This is most likely due to the wording of the second PHQ-2 item, which explicitly assesses feelings of hopelessness, thus likely over representing the hopelessness aspect of depression, compared to other depression measures.

### Limitations

As three items exhibited insufficient item-total correlations they were excluded from some analyses rendering a generalization of obtained results tentative. The huge number of models tested entails the risk of a coincidental fit to the data, rather than the confirmation of the “true” underlying model. Especially as the fit indices of several different models were virtually identical. Literature on the interpretation of model fit for ordered categorical variables is furthermore scarce. Hence, the interpretation heavily relies on findings generated from continuous data, the generalizability of which is questionable. Interpretation of results therefore has to remain tentative. Additional analyses applying item response theory could have provided further insight into item functioning.

## Conclusion

To the knowledge of the authors, this has been the first attempt to establish measurement invariance for the BHS by the means of multiple group CFA. (Iliceto, Fino, Sabatello, and Candilera [45] established measurement invariance regarding age in a larger model including the BHS, using a Likert scale and Iliceto and Fino tested for general model invariance in two random subsamples.) Measurement bias can lead to erroneous application and interpretation of cut-off scores, denying individuals in distress proper treatment [46]. Empirical findings could furthermore erroneously be generalized across groups. The establishment of cross cultural measurement invariance should hence become a priority to ensure comparability of results. Qualitative interviews concerning the subjective interpretation of the items #4, #8, #13, that did not seem to tap the construct well in the German sample, could help understanding the poor psychometric properties exhibited by these items. Regarding the overlap in item wording of the BHS and the PHQ-2, a validation in a German sample using a different depression measure might be appropriate.

## Additional file

Additional file 1: **Table A.** “Model fit of factorial solutions suggested in previous studies including the BHS”. (DOCX 26 kb)

## Abbreviations

BHS: Beck Hopelessness Scale
BSS: Beck Scale for suicide ideation
CFA: Confirmatory factor analysis
CFI: Comparative Fit Index
CI: Confidence interval
FLZ-8: Fragebogen zur Lebenszufriedenheit (Life satisfaction questionnaire)
KR-20: Kuder-Richardson Formula 20
MGCFA: Multi group confirmatory factor analysis
MTMM: multi trait multi method
PHQ-2: Patient health questionnaire 2
RMSEA: Root Mean Square Error of Approximation
SRMR: Standardized Root Mean Square Residual
TLI: Tucker Lewis Index
WHO: World health organization
WLSMV: Weighed least square means and variance adjusted estimation

## Footnotes

1 Table A in the electronic Additional file 1 shows the model fit of the models from the extensive literature regarding the BHS factorial structure.
