# Supplementary material for: Psychometric properties and measurement invariance of the Beck hopelessness scale (BHS): results from a German representative population sample
Source: BMC Psychiatry. 2018 Apr 25;18:110. doi: 10.1186/s12888-018-1646-6 (PMC5921745; doi:10.1186/s12888-018-1646-6)
Supplement: Supplementary file 1 — Table A. “Model fit of factorial solutions suggested in previous studies including the BHS”. (DOCX 26 kb) [file 12888_2018_1646_MOESM1_ESM.docx]

**Additional file**

**Table A** *Model fit of factorial solutions suggested in previous studies including the BHS*

| Author | Model | *χ2* | *df* | *CFI* | *TLI* | *RMSEA*  *[95% CI]* | |
| --- | --- | --- | --- | --- | --- | --- | --- |
| Onefactorial |  | 2205.127*** | 170 | 0.926 | 0.917 | 0.070  [0.067,0.073] | |
| Aish and Wasserman  (2001) |  | 45.863*** | 2 | 0.980 | 0.940 | 0.095  [0.072, 0.119] | |
| Two-factorial |  |  |  |  |  |  | |
| Bouvard et al. (1992) | capacity to imagine the future  (1, 3, 6, 8, 10, 11, 12, 13, 14, 15, 18, 19)  desire regarding the future  (2, 4, 5, 9, 16, 17, 20) | 1999.424*** | 151 | 0.923 | 0.913 | 0.071  [0.068, 0.073] | |
| Kao et al. (2012) | negative expectation of the future  (1, 2, 3, 4, 5, 6, 7, 13, 15, 18, 19)  loss of motivation for the future  (8, 9, 10, 11, 12,14, 16, 17, 20) | 2025.849 *** | 169 | 0.932 | 0.924 | 0.067  [0.064, 0.070] | |
| Nissim et al. (2010) | negative expectations  (1, 2, 3, 5, 6, 7,13,15, 18, 19)  loss of motivation  (4, 8, 9, 10, 11, 12, 14, 16, 17, 20) | 2002.155*** | 169 | 0.933 | 0.925 | 0.067  [0.064, 0.069] | |
| Pompili et al. (2007) | self-referent negative expectation of the  future  (11, 16, 17, 19, 20)  generalized negative expectation  (1, 7, 12, 14, 15, 18) | 732.840*** | 43 | 0.965 | 0.955 | 0.081  [0.076, 0.086] | |
| Steer, Beck, and Brown  (1997) | pessimism (1, 10, 15, 18)  resignation to the futility of changing the  future  (9, 16, 20) | 120.860 *** | 13 | 0.983 | 0.972 | 0.058  [0.049, 0.068] | |
| Tanaka et al. (1998) | doubt about a hopeful future  (1, 4, 5, 6, 8, 12, 13, 14, 15, 18)  belief about a hopeless future  (2, 7, 9, 10, 11, 16, 19, 20) | 1951.303 *** | 134 | 0.922 | 0.911 | 0.074  [0.072, 0.077] | |
| Three factorial |  |  |  |  |  |  | |
| Aloba et al. (2015) | negative expectations regarding the future  ( 9, 10, 11, 12, 13, 15, 17, 18, 19, 20)  positive expectations regarding the future  (1, 3, 5, 6, 8)  loss of motivation (2, 4, 7, 14, 16) | 1963.737*** | 167 | 0.934 | 0.925 | 0.066  [0.064, 0.069] | |
| Beck et al. (1974) | Feelings, about the future (1,5,6,13,15,19)  Loss of motivation (2,3,9, 11,12,16,17,20)  Future Expectations (4,7,8,10,14,18) | 1499.105 *** | 167 | 0.951 | 0.945 | 0.057  [0.054, 0.060] | |
| Dyce (1996) | expectation of success (1, 2, 3, 5, 6, 10, 13,  15, 19)  expectation of failure (9, 11, 16, 17, 20)  future uncertainty (4, 7, 8, 12, 14, 18) | 1762.634 *** | 167 | 0.942 | 0.934 | 0.062  [0.060, 0.065] | |
| Hill, Gallagher, Thomp-  son, and Ishida (1988) | hope (1,3, 5, 6, 13, 15, 19)  giving up (2, 8, 9, 11, 16, 17, 20)  plans about the future (4, 7, 10, 12, 14,  18) | 1621.650*** | 167 | 0.947 | 0.940 | 0.060  [0.057, 0.062] | |
| Rosenfeld et al. (2004) | positive future orientation (1, 3, 5, 6, 8, 10, 13, 15, 19)  tendency to not attempt to change  ones situation (2, 9, 16, 17, 20)  generally pessimistic outlook on  the future (4, 7, 11, 12, 14, 18) | 1001.225*** | 149 | 0.969 | 0.964 | 0.048  [0.046, 0.051] | |
| Steer, Iguchi, and Platt  (1994) | resignation (2, 9, 11, 16, 17, 20)  rejection (1, 6, 8, 13, 15, 19)  acceptance (4, 14) | 681.484 *** | 74 | 0.963 | 0.955 | 0.058  [0.054, 0.062] | |
| Steer, Kumar, and Beck  (1993) | rejection (1, 2, 3, 6, 7, 8, 10, 13, 15, 19)  acceptance (4, 12, 14, 17, 18)  resignation (9, 11, 16, 20) | 1593.562*** | 149 | 0.945 | 0.937 | 0.063  [0.060, 0.066] | |
| Niméus et al. (1997) | Loss of motivation (2, 7, 9, 11, 13, 14, 16, 20)  Expectations of the future (1, 10, 12, 15, 17, 18, 19)  Undefined (1, 2, 3, 6, 7, 11, 15, 19)  Undefined (4) | did not converge | | | | | |
| Bi-factor model |  |  |  |  |  |  |  |
| Szabó et al. (2016) | Content factor (all items)  Negatively worded items  (2,4,7,9,11,12, 16, 17, 18, 20)  Positively worded items  (1,3,5,6,8,10,13,15,19) | 27571.635*** | 190 | 0.985 | 0.981 | | 0.034  [0.031,0.036] |
| MTMM | Correlated traits  Correlated methods |  |  |  |  | |  |
| Boduszek & Dhingra (2016) | 3 trait factors  Feelings, about the future (1,5,6,13,15,19)  Loss of motivation (2,3,9, 11,12,16,17,20)  Future Expectations (4,7,8,10,14,18)  2 method factors  Negatively worded items  (2,4,7,9,11,12, 16, 17, 18, 20)  Positively worded items  (1,3,5,6,8,10,13,15,19) | 474.106*** | 146 | 0.988 | 0.984 | | 0.030  [0.027, 0.033] |

*Note.* CFI = Comparative Fit Index, RMSEA = Root Means Square Error of Approximation, TLI = Tucker Lewis Index; ***=*p*<.001.
